# Genetic distance predicts trait differentiation at the subpopulation but not the individual level in eelgrass, *Zostera marina*

**DOI:** 10.1002/ece3.4260

**Published:** 2018-07-03

**Authors:** Jessica M. Abbott, Katherine DuBois, Richard K. Grosberg, Susan L. Williams, John J. Stachowicz

**Affiliations:** ^1^ Center for Population Biology University of California Davis California; ^2^ Department of Evolution and Ecology University of California Davis California; ^3^ Institute for Wildlife Studies Arcata California; ^4^ Bodega Marine Laboratory Bodega Bay California

**Keywords:** functional diversity, genetic differentiation, genetic relatedness, phenotypic diversity, trait differentiation

## Abstract

Ecological studies often assume that genetically similar individuals will be more similar in phenotypic traits, such that genetic diversity can serve as a proxy for trait diversity. Here, we explicitly test the relationship between genetic relatedness and trait distance using 40 eelgrass (*Zostera marina*) genotypes from five sites within Bodega Harbor, CA. We measured traits related to nutrient uptake, morphology, biomass and growth, photosynthesis, and chemical deterrents for all genotypes. We used these trait measurements to calculate a multivariate pairwise trait distance for all possible genotype combinations. We then estimated pairwise relatedness from 11 microsatellite markers. We found significant trait variation among genotypes for nearly every measured trait; however, there was no evidence of a significant correlation between pairwise genetic relatedness and multivariate trait distance among individuals. However, at the subpopulation level (sites within a harbor), genetic (*F*_ST_) and trait differentiation were positively correlated. Our work suggests that pairwise relatedness estimated from neutral marker loci is a poor proxy for trait differentiation between individual genotypes. It remains to be seen whether genomewide measures of genetic differentiation or easily measured “master” traits (like body size) might provide good predictions of overall trait differentiation.

## 1 INTRODUCTION

Trait differences within and among species can influence ecological processes at multiple levels, from populations to ecosystems (e.g., Bolnick et al., 2011; Hector et al., 1999; Macarthur & Levins, 1967; Stachowicz, Kamel, Hughes & Grosberg, 2013; Wojdak & Mittelbach, 2007). However, identifying the traits that matter to ecological processes a priori is often challenging and context‐dependent (Naeem & Wright, 2003). Furthermore, measuring continuous trait variation among individuals within a species requires extensive effort and may not be practical for assemblages with a large number of taxa. Both of these challenges raise the question of whether measures of genetic distance can be used as proxies for functional divergence, based on the assumption that phylogenies, genealogies, or estimates of relatedness can reflect integrated phenotypic differences among taxa or individuals (Cadotte, Cardinale & Oakley, 2008; Felsenstein, 1985; Harvey & Pagel, 1991; Stachowicz et al., 2013).

Some evidence supports this assumption at the species level. For example, the outcomes of interspecific interactions can sometimes be predicted by the phylogenetic distinctiveness of the interacting species, with more closely related species competing more intensely for a narrower range of resources, leading to lower group productivity or greater probability of competitive exclusion (Burns & Strauss, 2011; Cadotte, Cavender‐Bares, Tilman & Oakley, 2009; Cadotte et al., 2008; Flynn, Mirotchnick, Jain, Palmer & Naeem, 2011; Maherali & Klironomos, 2007; Violle, Nemergut, Pu & Jiang, 2011). However, the underlying assumption that phylogenetic diversity serves as a proxy for trait differentiation is not always supported; in some cases, phylogenetic diversity influences ecosystem structure and functioning even when phylogenetic distance is not correlated with trait differences (Flynn et al., 2011; Tan, Pu, Ryberg & Jiang, 2012). Furthermore, not all traits relevant to the outcome of interactions are evolutionarily conserved (Best, Caulk & Stachowicz, 2013; Best & Stachowicz, 2013; Cavender‐Bares, Ackerly, Baum & Bazzaz, 2004; Cavender‐Bares, Keen & Miles, 2006; Moles et al., 2005; Silvertown, Dodd, Gowing, Lawson & McConway, 2006) and phylogenetic distance does not always influence ecological processes in the expected direction (Burns & Strauss, 2011; Cadotte, Davies & Peres‐Neto, 2017; Godoy, Kraft & Levine, 2014; Narwani, Alexandrou, Oakley, Carroll & Cardinale, 2013).

Similar approaches indicate that both trait diversity and genetic relatedness within species can influence the outcome of ecological interactions (Abbott & Stachowicz, 2016; Dudley & File, 2007; Stachowicz et al., 2013). For example, trait differences could lead to niche partitioning reducing competition among individuals and promoting coexistence (Chesson, 2000); alternatively, trait differences could lead to competitive exclusion if certain traits allow individuals to be competitively dominant in that habitat (e.g., Abbott & Stachowicz, 2016). Relatedness could influence intraspecifc interactions indirectly if it is correlated with trait differentiation (Jousset, Schmid, Scheu & Eisenhauer, 2011; Stachowicz et al., 2013) or directly through kin recognition (Aguirre, Miller, Morgan & Marshall, 2013; Dudley & File, 2007). However, the degree to which genetic relatedness serves as a reliable proxy for trait differentiation remains a key question. Within a species, genetic relatedness may not be tightly correlated with trait differentiation, especially where there is strong selection on ecologically relevant traits. In these cases, phenotypic differentiation often exceeds what might be predicted by genetic distance (McKay & Latta, 2002; Reed & Frankham, 2001). Intraspecific trait differentiation may also be correlated with genetic distance for some traits but not others, depending on the extent to which particular traits contribute to reproductive isolation (Wang & Summers, 2010). Similarly, drift‐based models of trait change suggest that the relationship between genetic distance and trait differentiation might be wedge‐shaped or saturating rather than linear (Cadotte et al., 2017). Furthermore, different metrics of genetic variation appear to have different effects on the outcome of ecological interactions (Abbott et al. 2017; Hanley, Hughes, Williams, Garland & Kimbro, 2016; Jousset et al., 2011), clouding the mechanistic interpretation of the effects of genetic variation in ecology.

**Figure 1:**
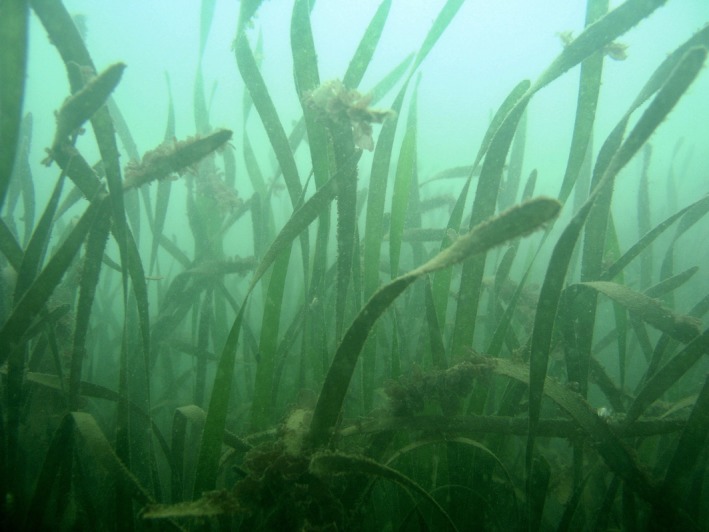
Eelgrass (*Zostera marina*) growing in Bodega Harbor, CA. Photograph by Jessica Abbott

In this study, we ask whether genetic relatedness can serve as a proxy for integrated trait differentiation within a species, at both the level of an individual and a subpopulation, using eelgrass, *Zostera marina*. Eelgrass is a foundation species that forms extensive meadows in bays and estuaries throughout the northern hemisphere, where it provides critical habitat for fishes and invertebrates, while buffering shorelines from erosion and playing a key role in nutrient cycling (Figure 1; Williams & Heck, 2001). Eelgrass reproduces sexually, as well as vegetatively, and genotypic diversity varies at scales of meters (1–15 genetically distinct individuals m^−2^ (hereafter “genotypes”) in Northern California, Hughes & Stachowicz, 2009), and among sites and tidal heights (Kamel, Hughes, Grosberg & Stachowicz, 2012; Olsen et al., 2004; Ort, Cohen & Boyer, 2012). Multiple genotypes potentially interact at an even finer scale, because as many as four unique genotypes can grow highly intertwined in a 10 cm by 10 cm area (J. Abbott and J. Stachowicz, unpublished data). Previous work shows that eelgrass genotypes differ in traits such as individual growth rate, nutrient uptake, susceptibility to herbivores, and detrital production (Hughes, Stachowicz & Williams, 2009; Tomas et al., 2011), and that these trait differences can predict assemblage performance (Stachowicz et al., 2013). Although much of the differentiation in traits observed in eelgrass across tidal heights is due to phenotypic plasticity (Dennison & Alberte, 1986; Li, Kim, Kim, Kim & Lee, 2013), there can be genetic differentiation in eelgrass growing at different depths (Kim et al., 2017; Ort et al., 2012), suggesting that there may be genetically based trait variation across depths. Trait differences can influence the intensity of competition between genotypes and patterns of coexistence, biomass accumulation, and population stability, with substantial consequences at the community and ecosystem level (Abbott & Stachowicz, 2016; Abbott et al., 2017; Ehlers, Worm & Reusch, 2008; Hughes & Stachowicz, 2004, 2011; Reusch, Ehlers, Hämmerli & Worm, 2005).

Our previous analyses of the covariance between genetic relatedness and trait differentiation in eelgrass suggested that the two were uncorrelated (Abbott & Stachowicz, 2016) or positively correlated, with more closely related individuals having more divergent traits (Stachowicz et al., 2013). However, these analyses used only six to eight genotypes collected from three sites at similar tidal heights and covered a narrow range of pairwise relatedness. The main goal of previous studies was to test the influence of genotypic richness or relatedness on intraspecific interactions, rather than to assess rigorously the relationship between genetic relatedness and trait differentiation. A larger, spatially stratified sample of genotypes provides a more rigorous test, while also allowing us to test the level of genetic and functional differentiation at the subpopulation level. Unfortunately, despite differentiation in important physiological and growth traits, these genotypes are not readily distinguishable morphologically, highlighting the importance of developing genetic proxies for ecologically relevant trait differentiation. Here, we quantify the genetic relatedness of 40 eelgrass genotypes collected from different sites and tidal heights within Bodega Harbor, CA, and measure a range of phenotypic traits in these individuals related to resource acquisition, morphology, growth rate, and competitive ability. We use these data to assess how trait variation is distributed among genotypes, sites, and tidal heights, and whether measures of genetic distance (both at the individual and subpopulation/site level) are reliable proxies for trait differentiation that could ultimately be used to predict ecosystem functioning better than simple genotypic diversity.

## 2 MATERIALS AND METHODS

### 2.1 Genotype collection

In May 2012, we collected 20 eelgrass ramets harvested at 2‐m intervals along a 40‐m transect at each of three tidal heights (high intertidal, low intertidal, and subtidal) at four sites within Bodega Harbor, CA. We collected an additional 20 ramets along a 40‐m transect from small (<4 m^2^) patches of eelgrass near the entrance to the harbor, which are all subtidal (see Supporting information Appendix A, for a map and GPS coordinates of collection locations). Bodega Harbor is a shallow harbor located 64 km northwest of San Francisco (38°19′25″N, 123°02′52″W). The harbor is about 5 km^2^ in area, much of which is mudflats at or above mean lower low water (MLLW) (NOAA Nautical Chart 18643, “Bodega and Tomales Bays”). Eelgrass grows in Bodega Harbor from about 0.25 m above MLLW in the intertidal to 3 m below MLLW in the deepest parts of the harbor adjacent to a dredged channel. The five eelgrass collection sites are distributed throughout the harbor, between 0.45 and 3.2 km apart. We transported the 260 eelgrass ramets collected from these sites to the Bodega Marine Laboratory (~2 to 4 km), where we trimmed the ramets to a single shoot with 3 cm of rhizome and 30 cm of leaf length and planted them in 11.4 cm diameter by 9.5‐cm high‐plastic flowerpots. We placed all pots in a single common garden flow‐through seawater tank; we randomly assigned the pots to an initial position and rotated the pot position weekly. We collected leaf clips from each ramet for genetic analysis.

### 2.2 Genetic analysis

We delineated genotypes and estimated relatedness using 11 microsatellite loci selected from a pool of >30 loci developed specifically for *Z. marina* (Abbott & Stachowicz, 2016; Oetjen, Ferber, Dankert & Reusch, 2010; Oetjen & Reusch, 2007; Reusch, 2000; Reusch, Stam & Olsen, 1999). We identified a total of 219 unique genotypes from the 260 ramets we collected. We estimated the relatedness of all genotype pairs with a regression‐based measure of the number of shared alleles, calibrated by the frequency of those alleles in the population, using the program STORM (Frasier, 2008). We estimated relatedness both using all genotypes from Bodega Harbor as a whole (allele frequency based on all genotypes and relatedness of all possible pairs estimated) and with genotypes from different subpopulations (based on *F*_ST_) independently (allele frequency determined for each subpopulation separately and relatedness between pairs within subpopulations estimated).

### 2.3 Population structure

To estimate the degree of genetic structure among sites and among tidal heights, we calculated Weir & Cockerham's F‐statistics (*F*_ST_) using ARLEQUIN 3.5.1.3 and tested for significance by 10,100 permutations of the data (Excoffier, Laval & Schneider, 2005). We used all 219 unique genotypes originally collected from the different sites in Bodega Harbor for these analyses, not just the 40 on which we measured traits.

### 2.4 Trait measurements

From the pool of 219 unique genets, we selected 40 genotypes to ensure that we included (a) a wide range of pairwise relatedness values and (b) genotypes from all tidal heights and sites (see Supporting information Appendix B, for multilocus genotypes and site/tidal height information). We transplanted these 40 genotypes into 3.79‐L plastic flowerpots and grew them in an outdoor common garden flow‐through seawater tank for the duration of trait measurements. We rotated pot position weekly to avoid position effects. The common garden tank was 4.5 m long and 1 m wide and held approximately 3,800 L of seawater. Seawater flowed into the tank via 10 inflow valves that were distributed along the length of the tank with a combined seawater flow rate of approximately 16 L/min.

**Figure 2:**
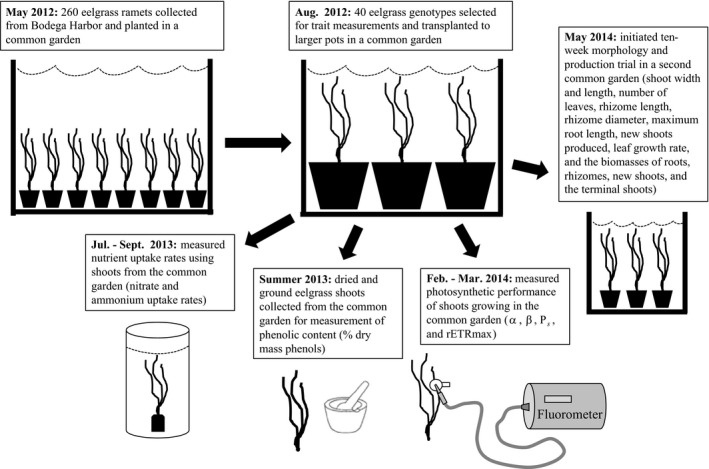
Diagram summarizing the work flow of the experiment from genotype collection to trait measurements. Text boxes describe each step, and any traits measured during a step are listed in parentheses in the text

We then measured a range of performance and resource acquisition traits for these 40 genotypes. We measured these traits only on new shoots produced while the plant was in the common garden, and not until shoots had acclimated to the common garden conditions for at least 10 months and had produced a minimum of three new shoots. Figure 2 summarizes the experimental design and timeline for genotype collection, propagation, and trait measurements.

### 2.5 Morphology and production

We harvested five shoots of each genotype from the primary common garden, and standardized each to a common module size of 3 cm of rhizome and 30‐cm shoot length, then planted them individually in 11.4 cm diameter by 9.5‐cm high‐plastic flowerpots. We did not standardize other traits within each module such as the initial number of leaves per shoot, shoot width, rhizome diameter, or maximum root length because these would have required significant damage to the modules that would have likely affected their survival and performance. The pots were placed in a second identical outdoor common garden in May 2014 at the beginning of the growing season. We started a second common garden so that we could retain the original genotypes in the primary common garden, while also destructively harvesting shoots for growth and morphological measurements. After a growth period of 10 weeks, we harvested the plants and measured shoot width and length, number of leaves, total rhizome length, rhizome diameter, maximum root length, new shoots produced, and the biomasses of roots, rhizomes, new shoots, and the terminal shoots. Because the transplanted modules used in the experiment came from plants which had been growing in a common garden for 2 years prior to the transplant, any differences among modules belonging to different genotypes are unlikely to be driven by differences in the environment in which they were raised. One week prior to harvesting plants, we punched holes in terminal shoots to measure leaf growth rate (Williams & Ruckelshaus, 1993).

### 2.6 Nutrient uptake rate

We measured biomass‐specific leaf nitrate uptake and root/rhizome ammonium uptake rates using two‐compartment chambers similar to the design in Terrados and Williams (1997). Eelgrass plants can absorb multiple forms of nitrogen in all their tissues; however, because nitrate is most available in the water column and ammonium in the sediments we measured nitrate uptake in leaf shoots and ammonium uptake in roots/rhizomes at ambient concentrations.

We collected eelgrass shoots from the primary common garden of 40 genotypes, cleaned them of all sediment, epiphytes, and invertebrates, and cut their rhizomes to 3 cm the day prior to allow wound healing. The roots and rhizomes of each shoot were compartmentalized from the leaf shoots by inserting the shoot through a slit in a watertight rubber stopper that was then inserted into a 40‐ml opaque plastic chamber filled with 35 ml of nitrogen‐free artificial seawater spiked with ammonium to 100 μM using a 5 M NH_4_‐N stock solution prepared with ammonium sulfate. We then placed the root/rhizome chambers with shoots inserted into 2‐L clear acrylic cylindrical chambers filled with 1 L of nitrogen‐free artificial seawater spiked with nitrate to 40 μM using a 2 M NO_3_‐N stock solution prepared with sodium nitrate. Sixteen acrylic chambers were seated in a water bath with water circulating through a chiller to keep chamber seawater temperature between 10 and 12°C (Bracken, Jones & Williams, 2011). To provide sufficient water flow to prevent mass transfer limitation of uptake, we attached submersible pumps to each chamber via inflow and outflow pipes. Full spectrum quartz halite lamps surrounding the water bath provided the chambers with photosynthesis‐saturating light (~700 μmol photons m^−2^ s^−1^).

We took water samples from the root/rhizome chambers and shoot chambers prior to the start of the experiment, then sampled the shoot chambers every hour for 4 hr, at which time we detached the root/rhizome chambers from the shoot chamber, removed the shoots, and took a final sample. We analyzed the shoot chamber samples (nitrate) using a Lachat 8000 series flow injection autoanalyzer and root/rhizome chamber samples (ammonium) using a Beckman Coulter DU640 spectrophotometer (Koroleff, 1976). After removing plants from the chambers, we divided them into shoots, roots, and rhizomes, and dried them at 60°C for at least 48 hr and then weighed each to obtain biomass‐specific uptake rates.

We ran uptake trials with between 10 and 14 genotypes per day and measured all genotypes each week for 9 weeks. Within a week, we randomly assigned eelgrass genotypes a day and position in the water bath. Some genotypes did not yield enough shoots in the common garden for the full set of nine replicates (one genotype had only enough shoots for three replicates, but most had seven to nine successful replicates).

### 2.7 Leaf phenolic content

Leaf phenolic content may be correlated with herbivore feeding preference on eelgrass (Buchsbaum, Valiela & Swain, 1984; Tomas et al., 2011; Vergés, Becerro, Alcoverro & Romero, 2007). We analyzed total phenolic content using approximately 4 mg of dried, ground leaf material from each genotype (pooled from three leaves) following a modified Folin–Ciocalteu method (see Bolser, Hay, Lindquist, Fenical & Wilson, 1998). We extracted phenolics with 2 ml of 80% methanol for 24 hr, and then quantified them with a spectrophotometer using caffeic acid as a standard. Ferulic and caffeic acids are two of the most abundant phenolics in *Z. marina* (Quackenbush, Bunn & Lingren, 1986; Vergeer & Develi, 1997), and previous work showed that caffeic, ferulic, or gallic acids standards for eelgrass phenolic content from shoots collected in Bodega Bay produced similar results (Tomas et al., 2011).

### 2.8 Photosynthetic rate

We evaluated the photosynthetic performance of each genotype using a Diving‐PAM^®^ (Pulse Amplitude Modulated) fluorometer (Walz, Germany) to measure maximum quantum yield (potential photosynthetic efficiency, *F*_V_/*F*_m_) and rapid light curves (RLC), which determine the effective quantum yield as a function of irradiance and can be used to assess light adaptation (Ralph & Gademann, 2005; Williams, Carranza, Kunzelman, Seema & Kuivila, 2009). First, we dark‐acclimated the outer leaves of each shoot for 30 min by placing a Waltz 4 mm opaque leaf clip 20 cm from the sediment surface on a leaf cleaned of epiphytes, then we immediately took maximum quantum yield and rapid light curve (RLC) measurements. RLCs comprised eight incremental steps of actinic light irradiance from 30 to 1,129 PAR (μmol photons m^−2^ s^−1^), and the resulting yield measurements were converted into a relative electron transport rate (rETR) using the following equation:

$$\text{rETR}=\Delta F/F_{m}^{\prime }*\text{PAR}*0.5*\text{AF},$$

where $\Delta F/F_{m}^{\prime }$ is the effective quantum yield, Δ*F* is the difference between background fluorescence *F* and *F* at each PAR increment, $F_{m}^{\prime }$ is the maximum fluorescence, 0.5 assumes that photons absorbed are equally distributed between photosystems I and II (Genty, Briantais & Baker, 1989), and AF is the standard absorption factor (0.55) for seagrasses (Durako, 2007).

To compare RLCs among genotypes we used curve fitting methods outlined in Ralph and Gademann (2005) to estimate characteristic parameters for each curve including: *α*, initial slope of the curve (rate of increase in photosynthesis with increasing light in light‐limited region of the RLC or photosynthetic efficiency); *β*, slope of the curve where yield declines (strength of photoinhibition); and *P*_s_, which is a scaling factor used to determine the maximum relative electron transport rate (rETRmax). We fit each curve to a double exponential decay function (Platt, Gallegos & Harrison, 1980) using the “nls” function in the stats package in R 3.0.2.

### 2.9 Trait distance metric

Using the data from our trait measurements, we calculated trait distances between all possible genotype pairs using standard methods (e.g., Petchey & Gaston, 2002). We used the data for the 17 traits that varied significantly between genotypes (all traits except for number of leaves and some photosynthesis parameters) to create a trait matrix in which trait values were standardized to have a mean = 0 and variance = 1. We then used the R “dist” function (R 3.0.3) to produce a Euclidean distance matrix of the multivariate trait distances between all genotype pairs for the 17 traits (hereafter referred to as “multivariate trait distance”). We did this for all pairs of genotypes within Bodega Harbor as a whole, as well as separately for genotype pairs within subpopulations, which were defined a priori by a sampling site being significantly differentiated from other sites based on *F*_ST_. As a second metric, we used a principal components analysis (PCA) to account for correlations among traits, and used the principal component scores for PC1 and PC2 to calculate the Euclidean distance between all pairs of genotypes (individual PC scores), and among genotypes from different sites or tidal heights (mean PC scores) in two‐dimensional trait space (hereafter “PCA trait distance”). We performed the PCA using scaled and centered (mean = 0 and variance = 1) trait values and used a correlation matrix to calculate principal components. The analysis was performed in R 3.0.3 using the prcomp function, which conducts the PCA using singular value decomposition of the data matrix. Separately, we assessed correlations among traits using pairwise regression (lm function from the stats package in R 3.3.3; R Core Team 2017).

### 2.10 Statistical analysis

We tested for variation among genotypes in each of the measured traits using separate ANOVAs in the “car” package in R. We analyzed the relationship between genotypic pairwise relatedness and trait distance (both multivariate trait distance and PCA trait distance) using a Mantel test (Vegan package) to account for nonindependence of the data caused by the presence of the same genotype in multiple pairwise combinations. We also used Mantel tests to evaluate the relationship between genetic differentiation (*F*_ST_) and trait differentiation (PCA trait distance) among genotypes from different sites and tidal heights within Bodega Harbor, CA. We also tested for a relationship between genetic differentiation (*F*_ST_) and geographic distance among sites. All analyses were performed using R 3.3.3 (R Core Team 2017).

Although Mantel tests (particularly partial Mantel tests) have been criticized for having low power and an inflated type I error rate (Bradburd, Ralph & Coop, 2013; Guillot & Rousset, 2013; Harmon & Glor, 2010; Oden & Sokal, 1992; Raufaste & Rousset, 2001), we believe that their use is justified in our analyses for the following reasons. First, we only use a simple Mantel test in our analyses, which suffers less from high type I error than partial Mantel tests (Guillot & Rousset, 2013; Harmon & Glor, 2010). Second, genetic variation in our system is not hierarchically clustered (as in a phylogeny), and thus, we have no reason to believe that null distribution of pairwise distances is structured in any way (which would violate an assumption of the Mantel test). Based on these considerations, we apply the Mantel test to assess significance of the correlation between trait and genetic distances.

To assess whether phenotypic differentiation among eelgrass from different subpopulations (sites) was greater or less than expected due to drift alone, we used *Q*_ST_‐*F*_ST_ analyses, where *Q*_ST_ is a measure of the quantitative trait differentiation among populations (calculated in a similar fashion to *F*_ST_), and the null expectation under drift is that *Q*_ST_‐*F*_ST_ = 0 (trait and genetic differentiation are equal). Significant deviation from the null expectation indicates that traits are under selection and local adaptation is occurring. We tested whether *Q*_ST_ was significantly different from the mean *F*_ST_ among subpopulations using a method developed by Whitlock and Guillaume (2009), which uses a parametric resampling approach (O'Hara & Merilä, 2005). In brief, this method predicts the null distribution of the difference between *Q*_ST_ and *F*_ST_ (*Q*_ST_‐*F*_ST_) using a mixture of parametric simulations and bootstrapping. The uncertainty of mean *F*_ST_ is modeled with a bootstrap across loci and a distribution of neutral *Q*_ST_ values is simulated using the estimated additive genetic variation and assuming the null hypothesis (that *Q*_ST_ = *F*_ST_) is true. The observed *Q*_ST_ values are then compared to the tails of the simulated null distribution. An extension of this procedure allows for the relatedness of offspring (individuals used to measure trait differentiation) to be specified (Gilbert & Whitlock, 2014). We specified offspring relatedness = 1 because we measured traits on clones of the same genotype. All analyses were performed using the R package *QstFstComp* (Gilbert & Whitlock, 2014) in R 3.3.3 (R Core Team 2017).

## 3 RESULTS

### 3.1 Trait variation among genotypes

Seventeen of the 21 traits differed among the 40 genotypes we measured for this study (ANOVA results in Supporting information Appendix C, trait means by genotype Supporting information Appendix D). Only the number of leaves per shoot and three of the photosynthetic parameters measured by PAM (*P*_s_, rETRmax, and β) showed no significant variation among genotypes.

**Figure 3:**
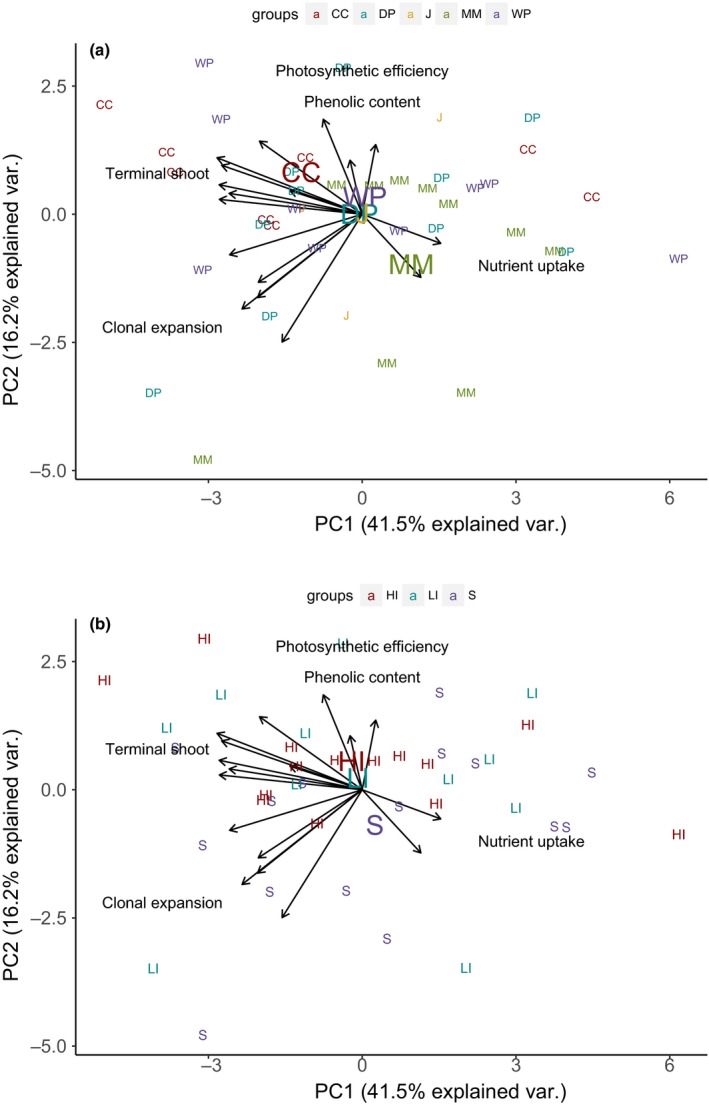
Principal components biplot depicting how the 40 genotypes differ in their traits across the two most significant principal components axes. The loadings for the 17 different trait measurements are represented by arrows, and the PC scores for the 40 genotypes are depicted by color‐coded labels (smaller font) for the site (a) or tidal height (b) from which the genotypes were collected (CC = Campbell Cove, DP = Doran Park, J = Jetty, MM = Mason's Marina, WP = Westside Park, HI = high intertidal, LI = low intertidal, S = subtidal). For ease of interpretation, instead of labeling all traits, major trait groupings are listed in the area where their arrows occur. “Nutrient uptake” includes both nitrate and ammonium uptake rates. “Clonal expansion” includes the mass and length of rhizome growth and number and mass of newly produced shoots. “Terminal shoot” includes morphological traits, mass, and growth rate of the terminal shoot. Color‐coded, larger font, site (a), or tidal height (b) labels are placed at the centroid of PC1 and PC2 scores for genotypes from each site or tidal height

The PCA using the 17 traits that differed significantly among genotypes showed that the 40 genotypes covered a broad range of trait space (Figure 3). The first two principal components accounted for 42% and 16% of the variation respectively. PC1 is positively correlated with ammonium and nitrate uptake rates and negatively with measures of plant size and clonal growth rate, whereas PC2 is positively correlated with measures of photosynthetic efficiency (*α*) and phenolic content, and negatively associated with traits related to clonal expansion. Higher order PCs contributed minimally to explaining variation in traits, and we did not consider them further. These patterns show that nutrient uptake rate and the size and growth rate of the terminal shoot are negatively correlated, whereas measurements of clonal expansion show a more orthogonal (independent) relationship with nutrient uptake rate and terminal shoot traits. Phenolic content and *α* also tend to show an orthogonal relationship with other traits (Figure 3). Pairwise correlations among traits corroborate the idea that nitrate and ammonium uptake rates are generally negatively correlated with many measures of plant size and growth rate, but interestingly, are uncorrelated with each other. In contrast, a wide range of plant size and growth traits are positively correlated with each other (Table 1).

**Table 1:** Pairwise trait correlations showing the correlation coefficients (first number listed) and linear regression *p*‐values (second number listed) for each combination

	Photosynthetic efficiency (*α*)	Shoot width	Shoot length	Rhizome diameter	Max root length	Leaf growth rate	Total rhizome length	Number of shoots	Terminal shoot mass	Rhizome mass	Root mass	New shoot mass	Leaf growth mass	Above: Belowground biomass	Phenolic content	Nitrate uptake rate
Shoot width	−0.03, 0.84															
Shoot length	0.15, 0.35	**0.53, <0.001**														
Rhizome diameter	0.12, 0.48	**0.80, <0.001**	**0.45, 0.003**													
Max root length	0.13, 0.40	0.27, 0.09	0.20, 0.21	0.22, 0.17												
Leaf growth rate	0.16, 0.33	**0.81, <0.001**	**0.63, <0.001**	**0.72, <0.001**	**0.45, 0.003**											
Total rhizome length	−0.10, 0.52	**0.35, 0.03**	0.01, 0.95	0.24, 0.13	0.17, 0.29	**0.34, 0.03**										
Number of shoots	−0.11, 0.51	**0.47, 0.002**	0.17, 0.29	**0.46, 0.003**	0.14, 0.41	**0.33, 0.04**	**0.53, <0.001**									
Terminal shoot mass	0.14, 0.39	**0.87, <0.001**	**0.73, <0.001**	**0.81, <0.001**	**0.44, 0.005**	**0.86, <0.001**	0.19, 0.23	**0.32, 0.05**								
Rhizome mass	0.009, 0.96	**0.60, <0.001**	0.19, 0.24	**0.58, <0.001**	0.28, 0.08	**0.58, <0.001**	**0.89, <0.001**	**0.59, <0.001**	**0.50, 0.001**							
Root mass	−0.07, 0.68	**0.65, <0.001**	**0.46, 0.003**	**0.54, <0.001**	**0.31, 0.05**	**0.72, <0.001**	**0.52, <0.001**	**0.61, <0.001**	**0.63, <0.001**	**0.68, <0.001**						
New shoot mass	−0.13, 0.43	**0.39, 0.01**	0.23, 0.14	**0.36, 0.02**	0.22, 0.18	**0.34, 0.03**	**0.67, <0.001**	**0.81, <0.001**	**0.31, 0.05**	**0.69, <0.001**	**0.65, <0.001**					
Leaf growth mass	0.08, 0.62	**0.80, <0.001**	**0.65, <0.001**	**0.74, <0.001**	**0.36, 0.02**	**0.92, <0.001**	0.21, 0.19	**0.32, 0.05**	**0.88, <0.001**	**0.46, 0.003**	**0.63, <0.001**	**0.33, 0.04**				
Above:belowground biomass	0.017, 0.92	0.23, 0.16	**0.49, 0.001**	0.21, 0.20	0.13, 0.41	0.12, 0.44	**−0.35, 0.03**	0.14, 0.38	**0.39, 0.01**	−0.28, 0.08	−0.11, 0.50	0.17, 0.30	0.30, 0.06			
Phenolic content	0.11, 0.50	−0.07, 0.65	0.09, 0.57	−0.06, 0.70	−0.01, 0.94	0.10, 0.52	**−0.35, 0.03**	−0.19, 0.24	0.03, 0.84	−0.25, 0.12	−0.02, 0.86	−0.28, 0.08	0.09, 0.60	0.02, 0.092		
Nitrate uptake rate (shoots)	−0.11, 0.49	**−0.34, 0.03**	**−0.31, 0.05**	**−0.38, 0.02**	−0.16, 0.31	−0.26, 0.10	−0.04, 0.79	**−0.49, 0.001**	**−0.40, 0.01**	−0.23, 0.15	−0.27, 0.09	**−0.37, 0.02**	**−0.37, 0.01**	**−0.37, 0.02**	−0.16, 0.33	
Ammonium uptake rate (roots)	−0.26, 0.10	**−0.32, 0.04**	−0.23, 0.16	**−0.32, 0.05**	**−0.42, 0.006**	−0.26, 0.11	0.18, 0.25	−0.18, 0.27	**−0.38, 0.02**	0.0004, 0.99	−0.23, 0.15	−0.10, 0.55	**−0.33, 0.04**	−0.24, 0.13	0.13, 0.44	0.19, 0.23

*Note*

All significant correlations are in bold. All significant and marginally significant positive correlations are highlighted in light gray and all negative correlations in dark gray.

We also analyzed trait differences as a function of the sites and tidal heights from which genotypes were collected to assess whether genetically based trait variation measured in a common garden was nonrandomly distributed in the field (i.e., evidence for local adaptation or environmental filtering). Out of the 21 traits, we found that shoot length differed significantly by site (*df* = 4, *F* = 4.94, *p* = 0.003, *R* ^2^ = 0.37) and maximum root length (*df* = 2, *F* = 8.5, *p* = 0.001, *R* ^2^ = 0.34) differed significantly by tidal height, when both site and tidal height were included in a model. We did not have enough replication to rigorously evaluate an interaction between site and tidal height.

### 3.2 Pairwise trait distance versus relatedness

**Figure 4:**
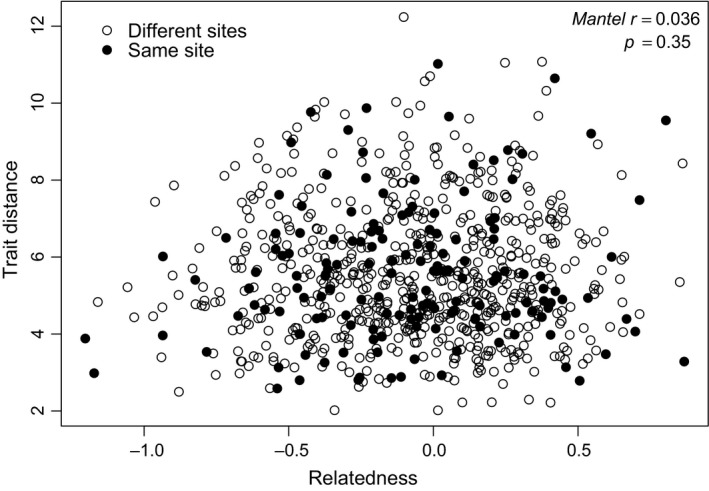
Relationship between trait distance and relatedness for all possible pairwise combinations of the 40 genotypes. Pairs of genotypes that were collected from the same site are depicted in filled in circles and pairs of genotypes collected from different sites are depicted in open circles

The multivariate trait distance we calculated for all possible pairs of the 40 genotypes was uncorrelated with their pairwise genetic relatedness (Mantel statistic *r* = 0.036, *p* = 0.351, Figure 4), regardless of whether we considered the entire sample, or partitioned the analyses according to their source subpopulation (Supporting information Appendix E). There was also no correlation between relatedness and trait distance for any of the 17 individual traits nor for different subsets of traits based on function (e.g., nutrient uptake and clonal expansion). This result did not change when we used the PCA trait distance as our measure of trait differentiation (Mantel statistic *r* = 0.0087, *p* = 0.459). Our two trait distance measures (multivariate and PCA) were highly correlated (*r* = 0.92, *p* = <0.0001).

### 3.3 Trait versus genetic differentiation across sites and tidal heights

**Figure 5:**
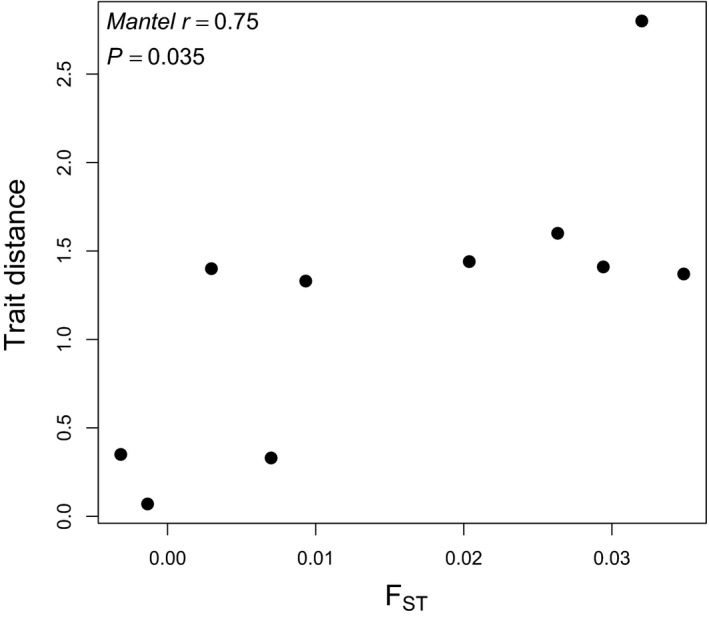
Positive relationship between multivariate trait distance and *F*_ST_ among pairwise site combinations in Bodega Harbor, CA

Using the position in trait space for each genotype represented in Figure 3, we estimated the mean trait composition of genotypes at each site or tidal height (centroid of genotypes from each location; Figure 3a,b). In contrast to the lack of correlation between genetic and trait differentiation at the individual level (pairs of genotypes), we found a positive correlation between genetic (*F*_ST_) and trait differentiation (PCA trait distance) across the sites from which the genotypes were sampled (Mantel statistic *r* = 0.75, *p* = 0.035, Table 2, Figure 5). We did not find any correlation between genetic and geographic distance across sites (Mantel statistic *r* = 0.41, *p* = 0.17). We found no evidence of a correlation between genetic and trait differentiation across tidal heights (Mantel statistic *r* = −0.77, *p* = 0.83), as trait and genetic variation among tidal heights was modest and with only three pairwise comparisons our power to detect patterns was low (Table 3).

**Table 2:** Trait (above the diagonal) and genetic (below the diagonal) differentiation for each site combination

	CC	DP	J	MM	WP
CC	—	1.40	1.44	2.8	1.33
DP	0.003	—	0.07	1.41	0.35
J	0.020	−0.001	—	1.37	0.33
MM	**0.032**	**0.029**	**0.035**	**—**	1.60
WP	0.009	−0.003	0.007	**0.026**	—

*Note*

Trait distance among the five sites measured as the two‐dimensional Euclidean distance between the mean PC1 and PC2 scores of each pair of sites (PCA trait distance) and genetic differentiation (*F*_ST_) among sites in Bodega Harbor, CA. Bold *F*_ST_ values are significant at *α* = 0.05. CC: Campbell Cove; DP: Doran Park; J: Jetty; MM: Mason's Marina; WP: Westside Park.

**Table 3:** Trait (above the diagonal) and genetic (below the diagonal) differentiation among the three tidal heights

	HI	LI	S
HI	—	0.36	1.32
LI	**0.012**	—	0.97
S	0.002	−0.002	—

*Note*

Trait distance among the three tidal heights measured as the two‐dimensional Euclidean distance between the mean PC1 and PC2 scores of each pair of tidal heights (PCA trait distance) and genetic differentiation (F_ST_) among tidal heights in Bodega Harbor, CA. Bold *F*_ST_ values are significant at *α* = 0.05, although the magnitude of the differentiation is low. HI: high intertidal; LI: low intertidal, S: subtidal.

*Q*_ST_‐*F*_ST_ analyses for sites detected no deviation from a null expectation that trait and genetic differentiation are caused by genetic drift alone. However, variation around the estimates of *Q*_ST_‐*F*_ST_ was quite high for all traits, likely due to low power in our analyses (Supporting information Appendix F).

## 4 DISCUSSION

A growing number of studies use genetic dissimilarity as a proxy for ecological differentiation (e.g., Cadotte et al., 2008; Stachowicz et al., 2013; Violle et al., 2011), often assuming a positive correlation between the two, yet there are few critical tests of the shape or strength of this relationship. We assessed whether neutral genetic differentiation among individuals and subpopulations of the eelgrass, *Z. marina*, at 11 microsatellite loci predicts their ecological differentiation with respect to a number of traits associated with resource acquisition and growth strategy. We measured these traits in a common outdoor tank, and found considerable variation in traits among eelgrass genotypes that was distributed among individuals, sites, and tidal heights. Genetic relatedness was uncorrelated with either differentiation in particular traits or multivariate trait distance indices. However, the magnitude of genetic differentiation at the site level did predict site‐level trait differentiation, although our sample size was relatively small (five sites). Thus, the correlation between genetic relatedness and trait differentiation among eelgrass genotypes appears to be scale dependent, just as has been found for correlations between phylogenetic distance and trait distance among species (Cavender‐Bares, Keen & Miles, 2006; Peay, Belisle & Fukami, 2011).

The lack of correlation between the genetic relatedness and phenotypic trait distance between two individuals corroborates our findings from a much smaller sample of six genotypes (Abbott & Stachowicz, 2016). Selection intensity and rates of evolutionary change can vary considerably among individual traits within a species (McKay & Latta, 2002), complicating the task of predicting general phenotypic differentiation from molecular differentiation at loci unrelated to the trait(s) in question. However, even considering each trait separately, we found no correlations between pairwise trait difference and relatedness. The lack of a relationship between pairwise relatedness and trait distance did not depend on whether the analysis pooled individuals across all sites or considered the unit of analysis to be sites that are genetically distinct based on *F*_ST_ (Supporting information Appendix E).

We used a relatively large number of highly polymorphic microsatellite markers to estimate relatedness, easing concerns about the accuracy of the estimates of relatedness as a general measure of genetic distance between individuals (Csilléry et al., 2006; Van De Casteele, Galbusera & Matthysen, 2001). Still, for polygenic traits determined by the additive effects of many loci (Le Corre & Kremer, 2003), similar phenotypes could be achieved by many different genetic combinations, obscuring a general relationship between pairwise genetic relatedness at a particular set of loci and overall phenotypic similarity. As genomic methods become more accessible, genomewide measures of genetic distance may prove to be better proxies for trait differentiation (Cadotte et al., 2017), but such data are as yet not available for eelgrass.

At larger scales, we found greater trait differentiation across sites than across tidal heights for traits measured in a common garden. The lack of consistent differences among plants collected from different tidal heights in Bodega Harbor suggests that observed trait differences across tidal heights in the field are primarily plastic responses to environmental conditions (see also Dennison & Alberte, 1986; Li et al., 2013). When measured in a common garden, the only trait that differed significantly among plants collected across tidal heights was rooting depth, with the deepest roots in the high intertidal, potentially an adaptation that enhances anchoring in the higher wave action of the intertidal. The lack of a correlation between genetic differentiation or relatedness and trait differentiation across tidal heights is not surprising given the limited genetic and phenotypic differentiation among tidal heights (Table 3). Other studies have found significant genetic differentiation across tidal heights in *Z. marina* at other locations (Kim et al., 2017; Ort et al., 2012), raising the possibility that genetically based trait differences in eelgrass from different tidal heights might occur in other populations.

The relationship between phenotypic and genetic differentiation that we observed at the site (or subpopulation) level could be explained by a combination of selection and genetic drift. Environmental differences across sites could create varying selection pressures that drive trait differences, while dispersal limitation among sites could reduce gene flow and allow divergence of neutral genetic markers. Alternatively, both traits and allele frequencies may diverge as a result of genetic drift due to low connectivity. Although we could not reject the null model of drift in the *Q*_ST_‐*F*_ST_ analysis, our power was low. The number of individuals with measured traits used for *Q*_ST_ estimates and the number of subpopulations tested were near or below the minimum sample size recommendations for a rigorous use of this statistical method (Gilbert & Whitlock, 2014). However, if neutral processes (drift) alone were responsible for the observed relationship among sites, then we would expect a similar positive relationship among individuals, which we did not find. Thus, it is still possible that the positive relationship between phenotypic and genetic differentiation is due to both drift and selection. Notably, the eelgrass genotypes from Mason's Marina (MM, see Supporting information Appendix A) were the most genetically and phenotypically distinct of our five sites (Figure 3), and MM is also the most environmentally distinct site (compared to the other sites, the sediment is more fine‐grained with higher organic content, water clarity is lower, and the sediment anoxic layer is shallower). Additionally, tidal currents at MM are weaker and water residence time is longer, potentially restricting genetic exchange with other populations. However, even when we removed Mason's Marina from the analysis, *F*_ST_ and trait differentiation remained positively correlated among the remaining four sites (Mantel test *r* = 0.67, *p* = 0.04), so this relationship is not entirely due to a single differentiated site.

The scale‐dependence of the within‐species relatedness versus trait differentiation relationship (a lack of relationship among individuals, but positive relationship among subpopulations) is analogous to the idea that the relationship between phylogenetic and trait differentiation among species depends on the phylogenetic scale under consideration (e.g., Cavender‐Bares, Keen & Miles, 2006; Peay et al., 2011; Stegen, Lin, Konopka & Fredrickson, 2012). Many of the issues we discuss for predicting within‐species ecological differentiation from genetic differentiation at neutral loci have analogies at the among‐species level. Convergent evolution can lead to higher trait similarity than expected in distant lineages, whereas character displacement can result in strongly divergent traits among closely related species (Dayan & Simberloff, 2005; Strong, Szyska & Simberloff, 1979). Furthermore, within a single clade, some traits may show a phylogenetic signal, whereas others do not (Losos, 2008), complicating the use of genetic proxies for overall differentiation in ecologically relevant traits (Cadotte et al., 2017). Thus, the predictive value of genetic relatedness for trait differentiation, both within and among species, likely only applies over a restricted range of genetic distances, which may vary among species.

Predicting pairwise trait differentiation among specific individuals remains an elusive challenge, despite the importance of such data for understanding competitive interactions and ecosystem functioning. Genetic data from a broader sample of the genome (e.g., SNPs) or at loci that actually control the measured traits could provide a stronger genetic proxy for trait differentiation at the among‐individual scale, but such data are currently unavailable for eelgrass. Independent of its relationship to traits, genetic relatedness in eelgrass influences the outcome of interactions, possibly as an indicator of the intensity of kin interactions (Abbott et al., 2017). Ultimately, as for interspecific comparisons (Cadotte, Albert & Walker, 2013), genetic and trait differentiation may provide complementary information about ecological interactions and outcomes (Jousset et al., 2011; Abbott et al., 2017). Alternatively, the strong positive correlations among many traits (Table 1) suggest that measuring relatively few simple traits might be a simpler proxy for overall trait differentiation. A better understanding of the mechanistic reason for these correlations would help assess the extent to which this is possible (Peiman & Robinson, 2017).

## CONFLICT OF INTEREST

None declared.

## AUTHOR CONTRIBUTIONS

JMA, JJS, SLW, and RKG conceived and designed the study. JMA and KD collected data for the study with assistance from SLW. JMA, KD, and JJS analyzed the data. JMA lead the manuscript preparation. All authors contributed critically to the drafts and gave final approval for publication.

## IN MEMORIAM: SUSAN WILLIAMS

Susan Williams, renowned marine biologist and devoted mentor, lost her life in a car accident in Petaluma, CA on April 24th 2018. Susan, a Distinguished Professor at the University of California, Davis, was a leader in ocean conservation, who focused on community engagement, science communication, and international collaboration to understand and protect marine ecosystems. Susan served as the director of the Bodega Bay Marine Laboratory from 2000 to 2010 and was an essential advisor to legislators, playing a key role in enacting legislation that expanded the boundaries of two national marine sanctuaries. Among many honors, in 2010, Susan received the UC Davis Academic Senate Distinguished Scholarly Public Service Award for her efforts in marine conservation. Susan was a pioneer in marine biology, paving the way for women to follow in her footsteps. She was a caring and dedicated mentor who used her success to support and empower her students. In 2009, she was honored by the UC Davis Consortium for Women and Research as an outstanding mentor for championing inclusion and diversity in the sciences. Susan's passion was inspiring, and the tremendous impacts of her mentorship, research, and leadership will continue her legacy.

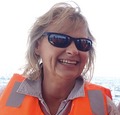

## Supporting information

 Click here for additional data file.
