# Supplementary material for: Genetic distance predicts trait differentiation at the subpopulation but not the individual level in eelgrass, Zostera marina
Source: Ecol Evol. 2018 Jul 3;8(15):7476–89. doi: 10.1002/ece3.4260 (PMC6106171; doi:10.1002/ece3.4260)

Appendix A: (A) Map of sites where the 40 genotypes were collected in Bodega Harbor, CA. MM = Mason’s Marina, DP = Doran Park, WP = Westside Park, CC = Campbell Cove, and J = jetty. Pins mark each end of a transect and there were 3 transects per site (expect at the jetty) at three different tidal heights: high intertidal, low intertidal, and subtidal (Google Maps, 2015). (B) Table with the GPS coordinates for the ends of each transect at the different sites and tidal heights, as well as the approximate tidal height of each transect relative to mean lower low water (MLLW).

**A**


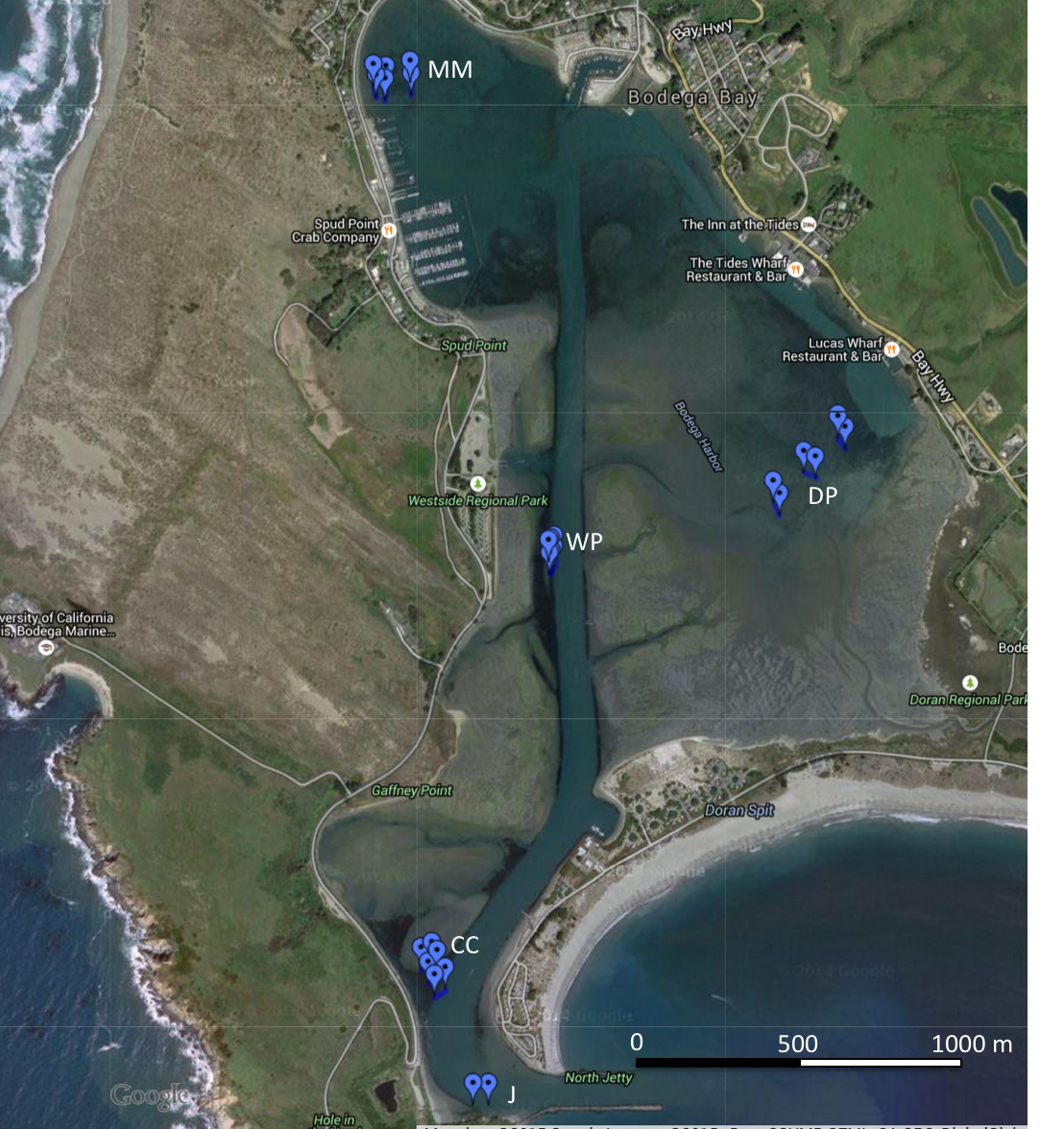


Google Maps. (2015). [Bodega Harbor, California] [Google Earth map]. Retrieved from:

<https://www.google.com/maps/@38.3232106,-123.0604582,3941m/data=!3m1!1e3!4m2!6m1!1sz_ptToqRYt6k.kMUcgphfy4wY?hl=en>

**B**

**
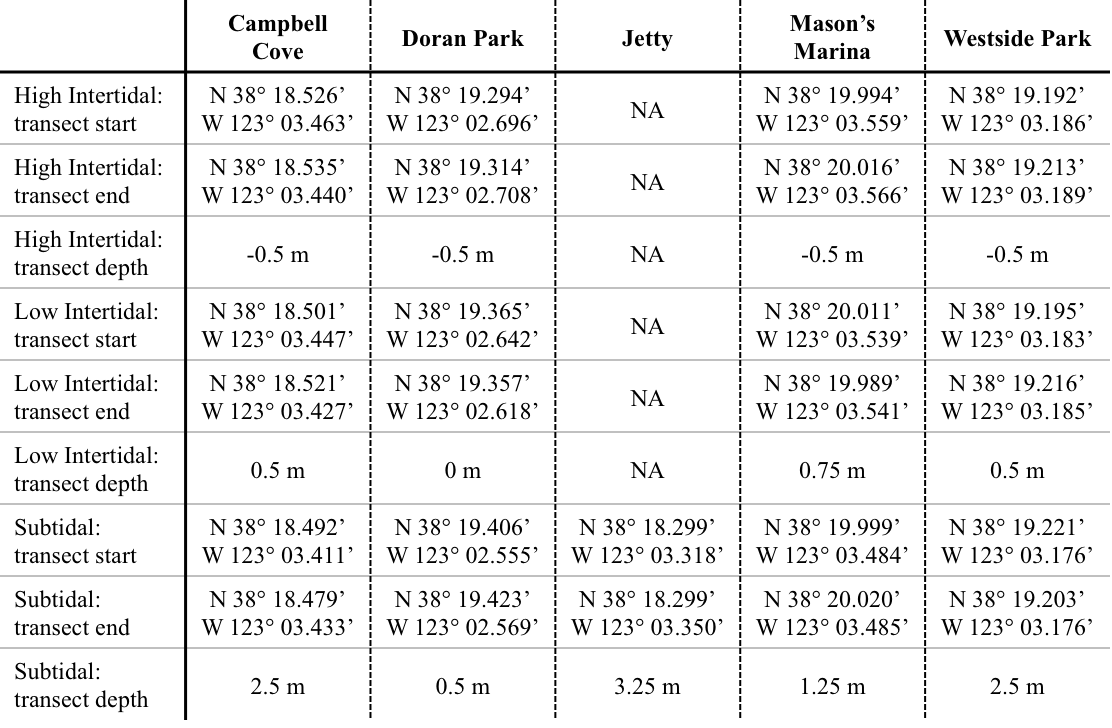
**

Appendix B: Multilocus genotypes for the 40 clones and which sites and tidal heights they were collected from. CC = Campbell Cove, DP = Doran Park, J = Jetty, MM = Mason’s Marina, and WP = Westside Park. HI = high intertidal, LI = low intertidal, and S = subtidal.

Appendix C: Table of degrees of freedom (DF), F-values, p-values, and R^2^ for trait differentiation among genotypes for the 21 measured traits. For traits marked with an asterisk there was a significant effect of the sampling week (nutrient uptake) or day (photosynthetic parameters) and the results that are presented include week/day in the model, however the R^2^ is for the proportion of variation explained by differences among genotypes when week/day are in the model, and not the proportion of variation explained by the model as a whole.

Appendix D: Table with the mean (*m*), standard deviation (SD), and sample size (n) for each trait by genotype. All mass measurements are in g, nutrient uptake rates are in uM/g/hr, phenolic conent is measured as percent dry weight, all morphological measurement as in cm expect for shoot width and rhizome diameter which are in mm, and leaf growth rates are measured as cm^2^/day.

Appendix E: Table and graphs of results from mantel tests of the relationship between pairwise relatedness and trait distance within subpopulation. Subpopulations were determined using the F_ST_ of all pairwise site combinations. Based on F_ST_ Mason’s Marina (MM) is genetically differentiated from all other sites, while Campbell Cove (CC) is differentiated from the jetty (J). All other site combinations show little differentiation, thus we looked at the relationship between pairwise relatedness and trait distance in within Mason’s Marina alone, Campbell cove alone, and Doran Park (DP), the jetty, and Westside Park (WP) together. We also looked at all of the sites except Mason’s Marina together. For each subpopulation we calculated relatedness using allele frequencies from that subpopulation alone.

Table of results from mantel tests:

Graphs of relatedness by trait distance for each subpopulation:

Appendix F: Estimates of Q_ST_ (genetically based quantitative trait differentiation) for each trait measured in common garden, calculated Q_ST_–F_ST_ where F_ST_ = 0.013, 95% confidence intervals for Q_ST_–F_ST_, and the P-value for the null hypothesis Q_ST_–F_ST_ = 0. Some Q_ST_ values are negative, which can result from the way Q_ST_ is calculated when it is close to 0. Negative Q_ST_ should be considered the same as zeros in terms of biological interpretation. We report 2-tailed P-values.


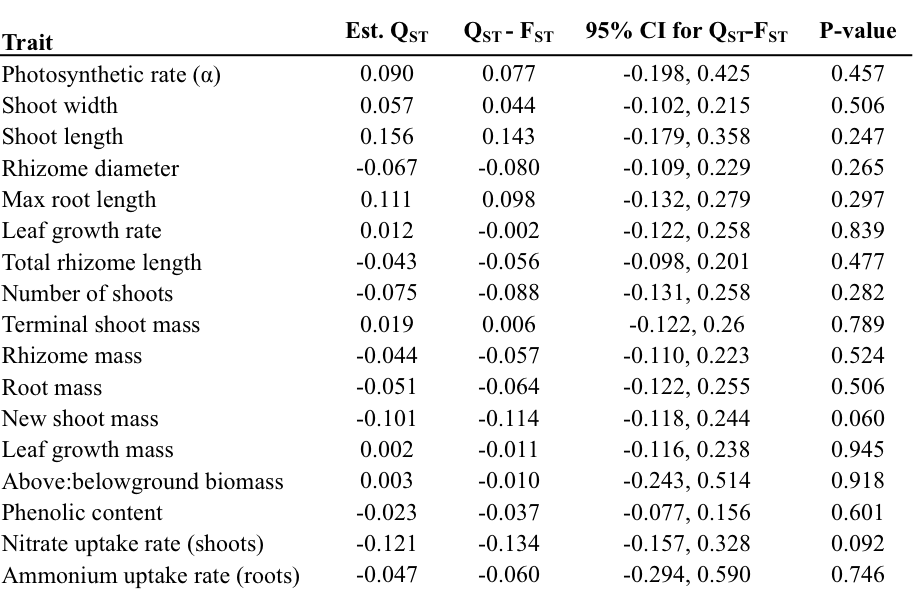

Supplement: Supplementary file 1 [file ECE3-8-7476-s001.docx]
